# “I'm Worth More than That”: Trait Positivity Predicts Increased Rejection of Unfair Financial Offers

**DOI:** 10.1371/journal.pone.0015095

**Published:** 2010-12-08

**Authors:** Barnaby D. Dunn, Dasha Makarova, David Evans, Luke Clark

**Affiliations:** 1 Cognition and Brain Sciences Unit, UK Medical Research Council, Cambridge, United Kingdom; 2 Behavioural and Clinical Neuroscience Institute, Department of Experimental Psychology, University of Cambridge, Cambridge, United Kingdom; University of Sheffield, United Kingdom

## Abstract

Humans react strongly to unfairness, sometimes rejecting inequitable proposals even if this sacrifices personal financial gain. Here we explored whether emotional dispositions - trait tendencies to experience positive or negative feelings – shape the rejection of unfair financial offers. Participants played an Ultimatum Game, where the division of a sum of money is proposed and the player can accept or reject this offer. Individuals high in trait positivity and low in trait negativity rejected more unfair offers. These relationships could not be explained by existing accounts which argue that rejection behaviour results from a failure to regulate negative emotions, or serves to arbitrate social relationships and identity. Instead, the relationship between dispositional affect and rejection behaviour may be underpinned by perceived self worth, with those of a positive disposition believing that they are “worth more than that” and those of a negative disposition resigning themselves to “taking the crumbs from under the table”.

## Introduction

Anyone who has witnessed siblings arguing over who rides in the front seat of the car can attest to the fact that humans are sensitive to fairness. Unfortunately our peers do not always treat us fairly, for example making a below market value offer for a property or proposing a less than reasonable pay rise, and we are forced to weigh up the competing demands of economic self interest versus social equity. How we respond to such inequity can have significant personal, social and economic consequences, and therefore it is important to understand the psychological mechanisms which regulate individual differences in our responses to unfairness. For example, in the current economic climate it would be valuable to understand and predict how workers in the broader economy will respond to pay freezes or cuts as the bonus culture returns to the financial world.

The tradeoff between financial self interest and equity is exemplified by economic choices in the Ultimatum Game (UG), where a proposer makes a once only offer of how to divide a sum of money, and a responder accepts or rejects the proposed division. If the offer is accepted, the proposal is implemented, but if the offer is rejected both players receive nothing. From a purely selfish perspective, the responder's ‘rational’ response is to accept all offers, no matter how unfair – after all it is ‘free money’. Nevertheless, most individuals reject inequitable UG offers [1].

In light of the realisation that decision making is driven by emotional as well as rational factors [2], ‘irrational’ rejection behaviour on the UG has been conceptualised as a failure of negative emotion regulation. Individuals experience anger in response to inequity, which is believed to override the economically sensible response of accepting whatever money is offered [3]. Principal evidence for this account is that activity in the right anterior insula, a brain area implicated in aversive emotions such as disgust and anger, is associated with greater rejection rates [4], that inducing a negative mood increases rejection [5], and that independent raters judge that participants are more angry following offers that they go on to reject [3]. Depletion of the neurotransmitter serotonin, which is implicated in emotional regulation, also leads to increased rejection after unfair offers [6]. Similarly, patients with frontal lobe lesions, who often show anger regulation difficulties, also display elevated rejection rates on the UG [7]–[8].

Such findings highlight an important role for *immediate* emotional reactions in accounting for departures from pure financial rationality in economic decisions. However, as yet, we know little about how longer-term emotional dispositions – whether we tend to experience broadly positive or negative emotions – impact on microeconomic behavior. A logical extension of the emotion regulation account is that those of a negative disposition will experience more state anger and therefore will reject a greater proportion of unfair offers. In contrast, those who are habitually positive will be less likely to reject in the face of inequity, due to a relatively low tendency to experience state anger. However, it is debated as to whether anger is best conceptualized as an approach emotion (more often associated with positive affect) or an avoidance emotion (more often associated with negative affect). Indeed, evidence increasingly links anger to activation of the approach system [9]. From this perspective, one would expect greater rejection behaviour in those of a positive as opposed to negative disposition. The primary aim of the present study was therefore to explore if dispositional affect relates to UG behaviour, and if any influence is mediated via alterations in state anger experience.

So far we have focused on proximate, mechanistic accounts of UG rejection behaviour (i.e. how affect at the time of the unfair offer triggers rejection). A different class of explanation attends to distal, functional consequences of rejection in the face of inequity. From a social perspective, it may be advantageous to reject unfair offers, as this helps preserve the individuals reputation as someone who cannot be ‘messed around with’ and because it provides negative social feedback to the proposer [10]–[11]. It is conceivable that trait affect could also interact with these social mechanisms. Those who are habitually positive may see themselves as having high social status that they need to preserve (leading to greater rejection in the face of inequity), whereas those of a negative disposition may see themselves as low in the social hierarchy and therefore will put up with unfair offers.

If trait emotion is functionally related to these social mechanisms, both positive affect (PA) and negative affect (NA) should show a stronger relationship to offers proposed by a ‘real’ person rather than offers randomly generated by a computer opponent, since the latter would not be relevant to the social hierarchy. To explore this possibility, the second aim of the present study was to examine the relationship between dispositional affect and responses to both human and computer generated unfair offers.

The final aim of the study was to examine whether dispositional emotions may underpin recent findings of a relationship between depression and reduced rejection of unfair offers on the UG [12]. At first glance this is a surprising result, given that depressed individuals show elevated state negative affect and impaired emotional regulation, and thus should reject more unfair offers. However, it has been reliably demonstrated that depression is also related to heightened dispositional NA and lower dispositional PA [13], and as discussed above elevated trait positivity may relate to increases in anger [9]. We also examine whether changes in UG rejection behaviour are specific to depression or a marker of more general psychopathology, giving the increasing realization that comorbidity between depression and related conditions like anxiety is the rule rather than the exception [14]–[15].

## Materials and Methods

The experiment was approved by the Cambridge University Psychology Research Ethics Committee, was conducted according to principles expressed in the Declaration of Helsinki, and participants gave written informed consent prior to taking part. Volunteers were given an honorarium of £6 per hour for their time and a contribution was made towards their travel expenses.

Participants (*N* = 44; 32 female; mean age = 37.75, *SD* = 17.28; mean estimated IQ = 118.68, *SD* = 8.82) were community volunteers recruited via an established departmental volunteer panel. They played the role of responder in 20 one shot UGs offering a division of £10. Prior to playing as UG responders, the participants were asked to propose five offers out of a £10 kitty and their photograph was taken. They were told that their photo and offers would be presented to future participants in the study. In turn, they were informed that the offers they would go on to receive had been made by prior volunteers in the study. This cover story was adopted to make it believable that the human offers that participants received were made by real people, and to enable examination of the relationship of PA and NA with proposal equity.

**Figure 1:**
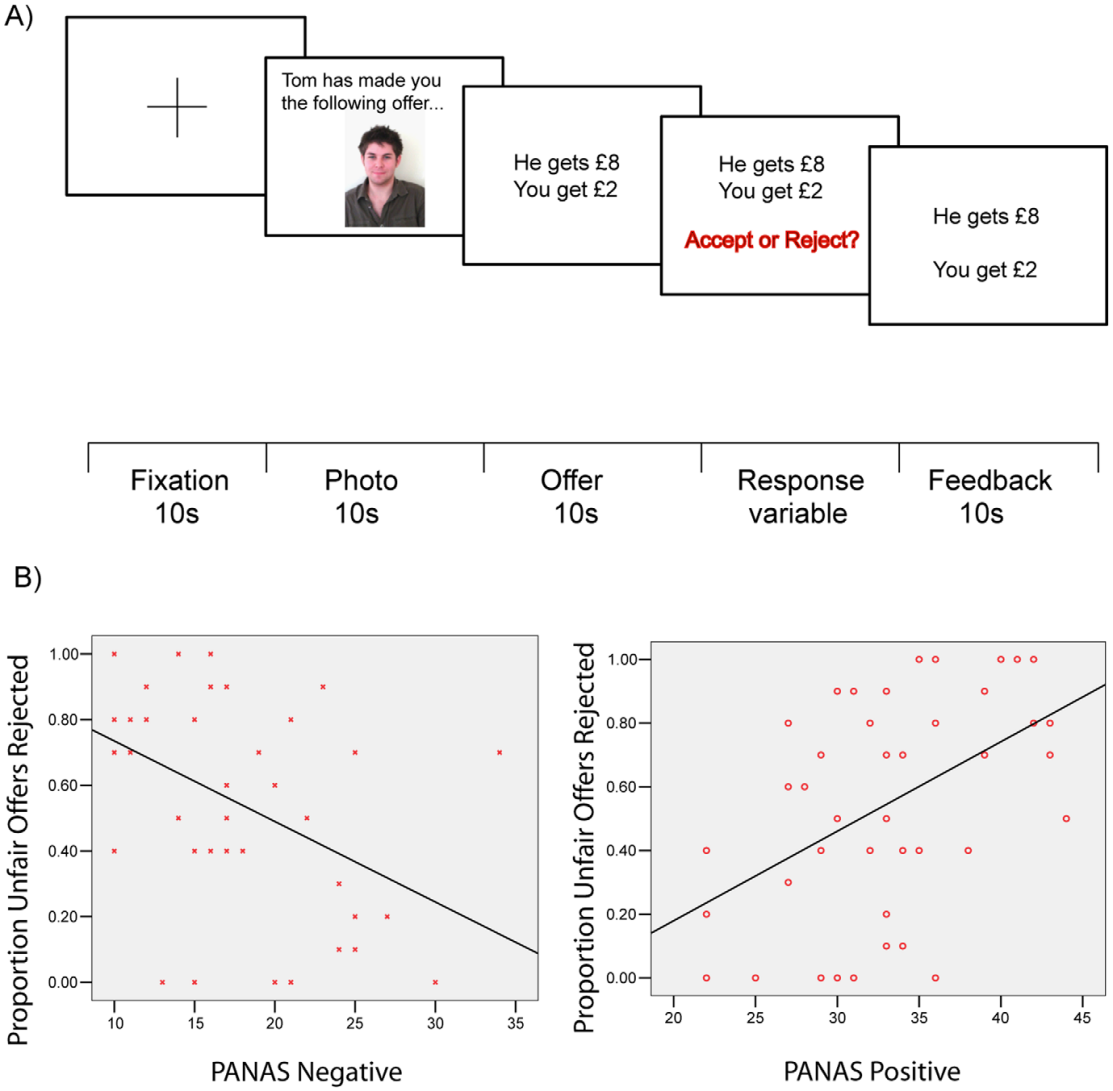
Overview of the Ultimatum Game (a) and the relationship of PA and NA with rejection behaviour (b). (**a**) On each of 20 trials participants attend to a fixation cross for 10 s, view a picture of the proposer or the computer for 10 s, the offer is presented for 10 s, and the participant then decides to accept or reject the offer. Feedback is presented for 10 s. (**b**) Relationship of PA and NA to the proportion of unfair offers that are rejected. As PA increases, participants reject more unfair offers. As NA increases, participants reject fewer unfair offers.

In the main task, there were 10 fair (£5) offers, 2 slightly unfair (£3), 4 moderately unfair (£2) and 4 very unfair offers (£1). Participants were instructed that half of each offer type were proposed by other people, and half were proposed by the computer (in reality, the offers were set by the experimenter and were identical across all participants) (see Figure 1a). To increase motivation, participants were instructed that one of their twenty responder decisions would be chosen at random to be paid out on. If they accepted this offer, both they and the proposer would receive the proposed shares of the pot. If they rejected this offer, neither player would receive any money.

After the UG task, participants rated how angry each offer type made them feel, on a scale from 0 (not at all) to 100 (extremely). We chose to rate anger retrospectively as we did not want to bias participants' behavioural decisions during the UG [3]. It is plausible that the expression of anger via a rating immediately post trial would serve to down-regulate anger to the extent that the participant no longer felt the need to reject unfair offers.

We measured dispositional positive and negative affect using the Positive Affect Negative Affect Scale (PANAS) [16]. This consists of 20 self-report items, with 10 measuring dispositional positive affect (PA; e.g. alert, strong, proud) and 10 measuring dispositional negative affect (NA; e.g. irritable, hostile, ashamed). For each adjective, participants rate to what extent they generally feel this way, on a scale from 1 (not at all) to 5 (extremely). PA and NA are viewed as relatively independent affective dimensions [13]. In the present sample, NA and PA were negatively related to one another, r = −.44, P<.−01. It has been argued that the phrase ‘affect’should be replaced with ‘activation’ in the PANAS, given that PA relates to an approach related, and NA to a withdrawal related, motivational system [17]. However, we continue to employ the term ‘affect’ as this is more widely used in the literature.

We additionally indexed levels of depression (using the Beck Depression Inventory; BDI-II) [18] and anxiety (using the Spielberger State-Trait Anxiety Inventory; STAI-T) [19]. Participants' full scale IQ was estimated using the National Adult Reading Test [20] to ensure they were in the normal intelligence range. The questionnaire measures were administered after the UG and another experimental task not reported here, to minimise any mood priming effects on behavioural performance. Table 1 reports the key UG and demographic variables for the sample.

**Table 1:** Summary of key study variables.

	Mean	*SD*
PANAS positive	33.18	5.67
PANAS negative	17.55	5.72
Age	37.75	17.28
Estimated Full Scale IQ	118.68	8.82
Beck Depression Inventory II	6.93	4.78
Spielberger Trait Anxiety	37.49	10.65
Human inequitable offer rejection rate	60%	37%
Computer inequitable offer rejection rate	50%	39%
Human equitable offer rejection rate	1%	5%
Computer equitable offer rejection rate	3%	15%
Proposals offered to future players	£4.60	£1.08
Anger to human inequitable offers	33.16	29.83
Anger to computer inequitable offers	21.53	26.76
Anger to human equitable offers	2.52	10.04
Anger to computer equitable offers	2.55	9.86

Anger rated on a scale from 0 (not at all) to 100 (extremely).

At the end of the task participants were fully debriefed about the deception elements of the design. Rather than receiving the product of one of their twenty gambles selected at random, participants instead all received an additional £5 reflecting a fair division of the money.

Alpha is set at .05 and all statistical tests are two tailed. As analyses are a priori motivated, no correction is made for multiple comparisons. All variables were visually inspected for normality prior to analysis and fell within acceptable statistical limits, Kolmogorov Smirnov>.01 in all cases. When exploring our first aim (mechanistic affect accounts of UG rejection) human and computer offers are pooled. When exploring our second aim (functional social accounts of UG rejection) we examine human and computer offers separately.

## Results

Consistent with previous results, participants rejected 55% (*SD* = 33%) of unfair offers on average. This was greater than rejections of fair offers (2%; *SD* = 9%), t(43) = 10.50, P<.001. Participants tended to reject more human inequitable than computer inequitable offers, t(43) = 1.96, P = .06. This effect was significant if restricted to the moderately unfair offers, t(43) = 2.14, P = .04, but not the slightly unfair, t(43) = 1.16, P = .25, or very unfair offers, t(43) = 1.35, P = .19. Inequitable offers were rated as inducing more anger than fair offers, t(43) = 7.18, P<.001, and this was more marked for human than computer offers, t(43) = 3.56, P<.01.

Table 2 reports correlations between dispositional affect and the UG task. Concerning our first aim, higher rejection of unfair offers was strongly related to lower trait NA, Pearson's r = −.42, P<.01, and greater trait PA, r = .48, P<.01 (Figure 1b). The relationship between unfair rejection rates and PA remained significant in partial correlations when controlling for NA, r_p_ = .36, P = .02, but the relationship between NA and unfair rejections was not statistically significant when controlling for PA, r_p_ = −.26, P = .09. This suggests that PA more strongly influences rejection behaviour than NA.

**Table 2:** Relationships between Ultimatum Game rejection rates, trait emotionality, and retrospective anger ratings.

	PA	NA	Anger unfair
Unfair rejections	.48**	−.42**	.09
Human unfair rejections	.50**	−.35**	.20
Computer unfair rejections	.34*	−.38**	−.03
Human fair rejections	−.03	.04	.17
Computer fair rejections	.03	−.10	.32*
Proposals	.05	−.21	.13
Anger unfair	.03	.36*	

Rejection rates are percentages of each offer type refused. Proposals  =  mean offers made to other players. Anger unfair  =  average retrospective anger ratings of human and computer unfair offers. Relationships are assessed using Pearson's correlation co-efficient. *  =  significant at P<.05. **  =  significant at P<.01.

To examine whether these dispositional effects were related to state anger effects we also examined retrospective anger ratings after unfair offers. Anger ratings of unfair offers were unrelated to PA, r = −.03, P = .86, but were significantly related to NA, r = −.36, P = .02. Crucially, the relationship of unfair rejection rates with both PA, r_p_ = .47, P<.01, and NA, r_p_ = −.43, P<.01, remained significant when controlling for anger ratings of unfair offers. There was also no interaction between state anger and either PA or NA in predicting rejection rates to unfair offers, Fs<1. While inequitable offer rejection rates were not simply related to anger ratings, r = .09, P = .55, this relationship was significant when controlling for NA, r_p_ = .29, P = .03, one tailed.

We also ran further analyses to examine if state anger mediated the relationship between dispositional affect and UG behaviour [21]. We used the SPSS procedure developed by Preacher and Hayes [22], which both computes the Sobel indirect test and provides a boot strapped estimate of the 95% confidence interval of the indirect effect. Mediation is indicated by a significant Sobel test and/or zero not falling within the bounds of the confidence interval. We additionally report the boot strapped estimate as this is more robust in the face of non-normality found in the distribution typically observed in small samples. State anger did not mediate the relationship between PA and unfair offer rejection, 95% confidence interval  =  −.004 to .004, Sobel indirect test Z<1, or the relationship between NA and unfair offers rejection, 95% confidence interval  =  −.0004 to .013, Z = 1.44, P = .15.

This replicates previous findings of greater state anger being related to rejection [3], but qualifies the effect by demonstrating its dependency upon trait negative affect. Crucially, the relationships between rejection behaviour and trait affect are independent from state affect. Indeed, the state and trait effects go in opposite directions to one another, with greater rejection being related to elevated state negativity but heightened trait positivity. Further, state affect did not significantly mediate the relationship between rejection and a positive or negative emotional disposition.

To explore our second aim, we looked at the relationship of dispositional affect to human and computer generated unfair offers. As discussed above, putative social mechanisms [10]–[11] should be more likely to exert an influence on decisions regarding offers proposed by ‘real’ players rather than those randomly generated by a computer. Therefore, if the effects of dispositional affect are playing out through social feedback mechanisms, the relationship of PA and NA with human unfair rejections should be the most marked. PA was significantly positively related and NA significantly negatively related to both human and computer unfair offer rejection rates, all Ps<.05. Next, we compared the magnitude of these correlations using the Williams test [23]. Crucially, there was no significant difference in the extent to which PA was related to computer (r = .34) versus human (r = .50) inequitable rejections, t<1, nor in the extent to which NA was associated with computer (r = −.38) versus human (r = −.35) inequitable rejections, t<1. Identical null results emerged if contrasting human and computer correlations for slightly, moderately, and very unfair offers in isolation, ts<1. Therefore, the relationship between dispositional affect and UG rejection behaviour is unlikely to be acting via social reputation and feedback accounts.

Next, we examined our third aim of whether PA and NA underpinned the associations previously reported between depression and reduced UG rejection [12]. As expected, depression severity was associated with lower PA, r = −.49, P<.01, and greater NA, r = .61, P<.001 [13]. Further, lower unfair rejection rates were related to higher depression levels, r = −.32, P = .04. When partialling out PA and NA this results was no longer significant, r_p_ = .03, P = .85. Moreover, these effects do not appear to be specific to depression. STAI trait anxiety was also related to lower PA, r = −.53, P<.001, greater NA, r = .80, P<.001, and lower rejection rates, r = −.49, P<.001. Again, the relationship between STAI trait anxiety and unfair rejection rates was no longer significant when partialling out PA and NA, r_p_ = −.16, P = .32. The depression relationship to rejection behaviour was no longer significant when controlling for trait anxiety, r_p_ = −.05, P = .74, while the anxiety relationship remained significant when controlling for depression, r_p_ = −.32, P = .04. This suggests that (in a non-clinical population) anxiety is more clearly related to rejection behaviour than depression symptoms, and that these relationships may be underpinned by differences in dispositional PA and NA.

We also attempted to rule out further alternative explanations of the data. First, the observed PA relationship could not be simply explained by greater sensitivity to fairness in more positively disposed individuals, since there was no relationship between ratings of the equitability of offers made to others in the proposal phase and either PA, r = .05, P = .77, or NA, r = −.21, P = .18. Further, statistically controlling for these offers did not alter the key relationship of unfair rejection rates to PA, r_p_ = .48, P<.01, or NA, r_p_ = −.40, P<.01. Second, given previous studies suggesting that testosterone influences rejection behaviour and that this is higher in men than women [24]–[26], we also examined whether there were gender effects on the UG. Neither mean proposals, total rejections, computer unfair rejections, PA or NA significantly differed between men and women, ts<1. However, men showed a non-significant trend to reject more unfair human offers than women, t(42) = 2.02, P = .05. Therefore, we explored a possible gender confound by repeating all analyses while additionally covarying for gender, and an identical pattern of results emerged. Third, we also found no significant relationship of proposal magnitude or rejection rates for any offer type with age or estimated full scale IQ, rs<.23, Ps>.15, suggesting these demographic variables cannot account for the present findings.

Finally, to investigate which aspects of PA and NA are particularly related to rejection behaviour, we correlated UG unfair offer rejections with individual item scores on the PANAS in exploratory analyses (see Table 3). Of the PA items, interested, strong, proud, determined and active were most strongly positively related to rejection behaviour. Of the NA items, distressed, scared, and afraid were most clearly negatively related to unfair offer rejection rates. Moreover, the case has previously been made that dispositional affect is related to self esteem [27]. For example, both PA and NA are uniquely related to self esteem measures [28], especially items that relate to self concept (e.g. ‘proud’, ‘strong’) [29]. Therefore, in additional analyses we pooled the subset of PA items related to self esteem by Brown and Marshall [29], and contrasted how clearly these were related to UG rejection behaviour compared to the remaining PA items. Unfair rejection rates were related to both the PA esteem items, r = .49, P<.001, and the other PA items, r = .36, P = .02. The correlation between the esteem PA items remained significant when controlling for the other PA items, r_p_ = .35,P = .02, but not vice versa, r_p_ = .05, P = .77.

**Table 3:** Relationships between individual PANAS items and rejection rates on the Ultimatum Game.

	Total unfair rejections	Human unfair rejections	Computer unfair rejections
Interested	.33*	.32*	.26+
Distressed	−.30*	−.29*	−.24
Excited	.18	.19	.12
Upset	−.26+	−.22	−.23
Strong	.43**	.51**	.25
Guilty	−.22	−.16	−.22
Scared	−.45**	−.36*	−.41*
Hostile	.00	.11	−.10
Enthusiastic	.25+	.30*	.15
Proud	.43**	.43**	.32*
Irritable	−.10	−.06	−.11
Alert	−.03	−.02	−.03
Ashamed	−.25+	−.20	−.25
Inspired	.31*	.23	.31*
Nervous	−.23	−.16	−.24
Determined	.31*	.34*	.21
Attentive	.22	.25	.14
Jittery	−.07	−.06	−.07
Active	.34*	.34*	.26+
Afraid	−.37*	−.28+	−.36*

Relationships are assessed using Pearson's correlation co-efficient. +  =  trend significant at P<.10; *  =  significant at P<.05; **  =  significant at P<.01.

## Discussion

Our findings demonstrate an important role for dispositional emotionality in accounting for microeconomic behaviour. Trait positivity (higher PA and lower NA) is related to greater rejection rates of unfair offers on the UG. This contrasts to state emotion effects, where induced negative emotion is associated with greater rejection rates [5]. These dispositional findings cannot be simply explained by existing emotion regulation [4] or social regulation [10]–[11] accounts of UG behaviour, since dispositional affect did not influence UG behaviour via fluctuations in state affect and showed no differential relationship to human versus computer unfair proposals.

The present results instead suggest a new explanation of UG rejection behaviour is required. We hypothesise that rejection behaviour is driven by internal self esteem. Individuals with high PA may feel they are “worth more than that” and therefore reject unfair offers. Individuals with high NA may feel they have no choice but to “take the crumbs from under the table” and therefore accept unfair offers.

Implicit in such an account is that rejection behaviour would serve an important self esteem regulation function, whereby rejecting an unfair offer maintains positive self regard and accepting an unfair offer lowers positive self regard. Therefore, there is self interest in rejecting an unfair offer, despite a monetary personal cost, in that it can help maintain a positive self image. As the fundamental function of this regulation is intra- rather than inter-personal, one would expect this to be equally the case for human and computer generated unfair offers. Clearly there would be important evolutionary consequences of deciding to “accept” or “reject” in some circumstances. For example, choosing whether to settle for a less than optimal life partner or risking up ending up alone in the hope that a superior mate will later be available.

These data may also explain altered social decision-making in clinical groups. The recent unexpected finding of decreased rejection rates in individuals with clinical depression [12] makes sense from the current account given that depressed groups typically show lower PA and higher NA [13]. However, this effect does not appear to be specific to depression, in that in the present sample a relationship was also found between trait anxiety and reduced rejection rates. Moreover, the depression relationship did not hold when controlling for anxiety, whereas the anxiety relationship did hold when controlling for depression. Of course, this conclusion needs to be interpreted tentatively, given the non-clinical nature of the present sample and requires replication in those who are clinically anxious and depressed.

It is important to emphasise that our self esteem proposal is in no way mutually exclusive to existing state affect or social accounts of UG rejection. We simply show that the effect of trait affect on UG rejection is independent of these other mechanisms. In our view, it is likely that performance on the UG is multiply determined by state affect, trait affect, social factors and a range of other mechanisms. Individuals could plausibly differ with regard to which of these mechanisms most influence their decisions. Moreover, different situations may lead to one mechanism being more salient than others. For example, when being watched or when feedback will be delivered to the proposer, social factors may come to the fore. Following a strong mood induction, state anger factors may instead be more influential. Future research could usefully examine how individual difference variables and manipulating parameters of the UG in these ways influences different underlying decision-making mechanisms.

There are a number of limitations to the present study which need to be held in mind. First, we did not directly measure self esteem using an established experimental measure. While the PANAS has been previously associated with self esteem and inspection of individual items showed the highest loading items were self-referent, our self esteem hypothesis nevertheless remains tentative until replicated with a specific measure of this construct. In particular, here we measure the affective but not cognitive components of self esteem [27]–[28]. Second, we rated anger retrospectively to avoid biasing rejection decisions. However the cost of this choice is that it introduces potential recall biases into the data and may reduce the possibility that anger ratings will be related to choice behaviour. We chose not to use the open ended questions (e.g. ‘how do you feel’) after each trial employed by Pillutla & Murninghan [3], as these might also have systematically under-estimated anger due to their lack of specificity. Further, participants may have used the ratings as a way of venting anger and therefore reject less. Retrospective ratings have been used in other studies, presumably for similar reasons [8]. It will nevertheless be important to establish if similar results hold when taking trial by trial state affect ratings. Third, completing the UG may have then biased how participants filled in the questionnaire measures. This seems plausible for state measures of mood. However, we feel this is less likely with the measures used here. The STAI-T and PANAS are trait (dispositional) measures and the BDI-I measures mood over the past week.

In summary, rejection to unfair offers on the UG is related to increased positive and decreased negative trait affect, supporting a new self esteem account of individual differences in microeconomic behaviour. There may be contemporary real world parallels to the findings reported here. In the current economic climate, many individuals in the broader economy are facing the prospect of pay freezes or cuts, despite the bonus culture returning to the banking world. In extreme cases, this has led to strikes and rioting in Greece when stringent austerity measures were introduced. The current account would predict high self esteem may make individuals more likely to ‘walk’ when confronted with such inequitable situations.
